# Rapid Point-of-Care Testing for Detection of HIV and Clinical Monitoring

**DOI:** 10.1155/2013/287269

**Published:** 2013-05-23

**Authors:** D. R. Arora, Megha Maheshwari, B. Arora

**Affiliations:** ^1^Department of Microbiology, SGT Medical College, Budhera, Gurgaon, Haryana 123505, India; ^2^Shri Guru Gobind Singh Tricentenary Medical College, Village Budhera, Gurgaon, Haryana 123505, India; ^3^Department of Pathology, SGT Medical College, Budhera, Gurgaon, Haryana 123505, India

## Abstract

Reversing and arresting the epidemic of HIV are a challenge for any country. Early diagnosis and rapid initiation of treatment remain a key strategy in the control of HIV. Technological advances in the form of low-cost rapid point-of-care tests have completely transformed the diagnosis and management of HIV, especially in resource limited settings, where health infrastructure is poor and timely access to medical care is a challenge. Point-of-care devices have proven to be easy to transport, operate, and maintain, and also lower-skilled staff is equally able to perform these tests as compared to trained laboratory technicians. Point-of-care tests allow rapid detection of HIV allowing for rapid initiation of therapy, monitoring of antiretroviral therapy and drug toxicity, and detection of opportunistic infections and associated illnesses.

## 1. Introduction

Testing and treatment are key elements in the effort to control HIV, and testing services are rightly considered as the gateway to the treatment facilities. Data suggest that HIV-infected individuals who are aware of their status are more likely to adopt risk reduction behaviour than those who are not as discussed by Higgins et al. [1]. With a diagnosis of AIDS, consideration may be given to the initiation of antiretroviral treatment, which reduces viral load and infectivity as discussed by Rotheram-Borus et al. [2]. From a public health perspective, it is advisable to recommend testing to those at risk for HIV and to make testing easily accessible. The idea is to detect every HIV positive whether it belongs to high risk group, a pregnant woman, or a patient of tuberculosis or reproductive tract infection approaching the health system for health needs and refer him/her to the nearest antiretroviral therapy (ART) centre. Providing quality laboratory services for HIV testing to all those who need it is a challenging task.

## 2. Point-of-Care Tests for HIV

Point-of-care (POC) testing of HIV refers to the practice undertaken by health care professionals of providing pretest counseling, posttest counseling, and a preliminary HIV antibody result at the time of testing outside of a designated laboratory. The standard methods of HIV testing (enzyme linked immunosorbent assay (ELISA) or western blot with confirmatory testing using p24 antigen detection or viral nucleic acid detection) can take several days for result availability as discussed by Arora et al. [3]. A significant proportion of individuals who agree to undergo HIV serologic testing do not return to the HIV testing site to receive their test results as discussed elsewhere [4–6]. POC testing of HIV attempts to address delay in detection of HIV status by providing preliminary antibody results. POC tests can be most useful in resource limited settings (RLS) or outreach settings where there is lack of well-trained laboratory technicians, poor physical infrastructure, extremes of climate, and lack of uninterrupted power supply, all of which impact the use of laboratory technologies. 

Rapid HIV test kits are designed to test for HIV antibodies. These deliver results within about 20 minutes of a specimen being taken; so, results are available within a single consultation. Rapid test devices (RTDs) are typically capillary flow tests for use on whole blood (e.g., fingerprick), plasma, urine, or oral fluid as discussed elsewhere [7–10]. They detect HIV antibodies against HIV 1 and 2 antigens produced by oligopeptide synthesis or recombinant DNA technology. Quick turnaround time, ease of sampling, performance and reading results, no requirement of cold chain, and specialized equipment make these tests highly suitable in RLS. Since, oral fluid/saliva testing is more convenient, noninvasive and safe for laboratory workers, it can serve as an alternative for screening as well as surveillance purposes as discussed by Garg et al. [11]. Oral fluid sampling for HIV could particularly benefit the uptake among children and injectable drug users who may have collapsed blood vessels. However, not all RTDs are usable at the point-of-care (e.g., they require serum separation but still give results in a few minutes). Any HIV POC test approved for use is required to have sensitivity and specificity equivalent to HIV screening test kits (ELISA) approved for laboratory use as discussed by Shott et al. [12]. Currently, seven FDA approved HIV RTDs are available in the market [13]. 

The field has also advanced with the development of over-the-counter (OTC) self-testing options for HIV and multiplexed platforms that allow for simultaneous detection of infections associated with HIV, such as hepatitis B and C and syphilis. Researchers believed that home testing could be valuable “in empowering individuals to manage their HIV risks; in helping couples to learn their partners' HIV status before the initiation of sexual relations; and in addressing the three principal barriers to wider HIV-test acceptance: stigma, convenience, and privacy” as described by Walensky and Paltiel [14]. FDA approved the OraQuick In-Home HIV Test, the first over-the-counter home use rapid HIV test kit to detect the presence of antibodies to HIV-1 and HIV-2 [15]. 

Fourth generation HIV RTDs that detect both antigen and antibodies (ARCHITECT HIV Ag/Ab Combo Assay, Alere Determine HIV 1/2 Ag/Ab Combo assay) are being developed. They allow for early detection of HIV infection, prior to the emergence of HIV antibodies, therefore reducing the window period of antibody detection [16]. These tests, however, need validation and extensive performance evaluation in diverse field settings.

## 3. When to Use the HIV POC Test Kits

POC testing of HIV is not designed for screening the general population; it is to be used to screen patients at high risk for HIV. The rapid turnaround time associated with its use can guide urgent decision making. This makes it suitable for use in targeted clinical scenarios where the immediate administration of antiretroviral drugs is recommended to reduce the risk of transmission or in cases where the patient's management may be altered by the availability of a reactive test result. 

### 3.1. Obstetric Settings

Testing pregnant women for HIV at the time of labor and delivery is the last opportunity for prevention of mother-to-child HIV transmission (PMTCT) measures, particularly in settings where women do not receive adequate antenatal care. However, HIV testing and counseling of pregnant women in labor is a challenge, especially in resource-constrained settings. In India, many rural women present for delivery without any prior antenatal care. Those who do get antenatal care are not always tested for HIV, because of deficiencies in the provision of HIV testing and counseling services as discussed elsewhere [17, 18]. POC testing should be provided to women with risk factors for HIV infection, but no recorded HIV status presenting in established labour as access to immediate HIV results improves the judicious use of antiretroviral prophylaxis as discussed by Cohen et al. [19].

### 3.2. Blood and Body Fluid Exposure/Health Care Worker Occupational Exposure

Knowledge of the source of the individual's HIV status during an evaluation of blood and body fluid exposure can help to determine more precisely those situations where HIV prophylaxis might be useful. HIV POC testing of source individuals offers an opportunity to eliminate anxiety and the unnecessary use of postexposure prophylaxis in the exposed person.

### 3.3. Acutely Ill Patients

In some clinical situations, it may be critical to have a rapid HIV diagnosis so that immediate and appropriate therapy or further diagnostic work-up can be provided, for example, a patient with risk factors for HIV who presents with pneumonia for which differential diagnosis would include *Pneumocystis jirovecii *pneumonia or patients undergoing hemodialysis.

### 3.4. An Individual at High Risk for HIV Acquisition

Consenting patients who are at high risk for HIV (needle stick source, from endemic area, injectable drug users, sexual partner with AIDS or positive HIV, history of unprotected sexual intercourse, multiple sex partners, sex partner of high risk person(s), sex worker, and homosexual men) and who have not had an HIV test in the previous 3 months, or who are unaware of their HIV status as discussed by D. R. Arora and B. Arora [20].

### 3.5. Patients Attending Sexually Transmitted Diseases (STD) Clinics

POC testing is acceptable, feasible, and leads to timely entry of people with HIV positive tests into the health care facility as discussed by Kendrick et al. [21]. Also among the STD clinic attendees presenting with genital ulcer, HIV reactivity (4%) was found to be statistically significant as discussed by Arora et al. [22].

## 4. Challenges in POC Testing

### 4.1. Counseling

POC HIV testing requires pre- and posttest counseling to be modified from the usual HIV counseling that accompanies standard HIV testing. However, certain clinical situations may make detailed pretest counseling difficult, for example, rapid testing for pregnant women in labour. In these situations, informed consent for testing is a minimum requirement. Informed consent is a process of communication that enables a person to make a reasonable and informed decision. Pretest counseling is critical in preparing patients for the implications of the test, and in cases of reactive test results, ensuring that they return for confirmatory test results. Posttest counseling provides information on risk behaviour, potential links to community resources, and rationale for behaviour changes needed to reduce risk. In the case of a nonreactive test result, the counseling session provides the opportunity for an exchange of information on the individual's perceived risks and cofactors and on harm reduction and prevention. Patients with risk activities in the last 3 months may not have detectable antibodies at the time of the test. These patients should be counseled as to the need for repeated HIV testing and on the need to protect their partners when engaging in high risk activities. With reactive test results, the implications are just as great in terms of reduction in risk of transmission, provision of health care information, and referral to ART centres. 

### 4.2. Quality Assurance

In contrast to the situation in standard HIV testing, the health care workers in the POC setting assume responsibility for specimen collection, testing, and counseling of the patient. Adequate resources, appropriate training, and the implementation of quality assurance practices will be critical in ensuring the proper administration of the test and the correct interpretation of the test result. 

### 4.3. Regulatory Approval

Rapid HIV test kits must be licensed for use in the country. 

### 4.4. Performance Characteristics of Rapid Test Devices

RTDs are generally satisfactory for the detection of uncomplicated HIV infection (or its absence) but are less sensitive than lab-based ELISAs and automated systems for detecting early infections (seroconversion). Also specificity of RTDs is lower than conventional ELISAs although it can be improved by immediate repeat of all RTD positives as discussed by Kagulire et al. [23]. Since these may not be reliable in the “window” period and appropriate repeat testing should be advised, no currently available RTDs incorporate HIV p24 antigen detection in contrast to the commonly used conventional combination ELISA laboratory tests which are more sensitive in early HIV infection.

### 4.5. Ethical Implications

Ease of testing might lead to people being tested without their voluntary, specific, and informed consent. This is a particular risk where patients are anaesthetised (e.g., occupational exposure) or unable to communicate (e.g., woman in labour) or otherwise lack capacity to make decisions. 

### 4.6. Staff Training

Appropriate training on the use of kits, reading of results, detection of errors, quality assurance, counseling, and regular assessment of staff who will be performing POC testing is required for providing point-of-care testing as discussed by Kabra and Kanugo [24].

### 4.7. Patients with a (Preliminary) Positive Test

A small number of people who are not HIV infected will produce a positive (reactive) result when tested with an HIV antibody test kit, including the rapid test. Because of this, all reactive test results must be confirmed using a laboratory-based confirmatory test. The importance for confirmatory testing at an approved HIV testing laboratory needs to be emphasized to rule out the possibility of a false-positive result in the rapid HIV test and to confirm a true positive result. 

## 5. Cost-Benefit Evaluation of HIV POC Testing

Costs and adverse events associated with the use of HIV POC tests (labour, training, test kit, and cost of antiretroviral medication in response to false reactive result) will be compared to the cost and adverse events associated with untreated HIV infection resulting in transmission to the newborn (among women with no prenatal testing), use of antiretroviral medications when not required (in cases of occupational exposure), and increased morbidity and mortality associated with delay in HIV status detection of an acutely ill patient. The cost-effectiveness of routine HIV screening in health care settings, even in relatively low-prevalence populations, is similar to that of commonly accepted interventions, and such programs should be expanded as discussed by Sanders et al. [25].

## 6. Clinical Monitoring of Patients

Access to antiretroviral therapy (ART) has increased dramatically over the past decade in low- and middle-income countries. However, successful management of HIV requires patients receiving ART to be monitored routinely to assess treatment efficacy and detect treatment failure due to drug resistance. The standard of care to monitor ART is quantitative viral load testing based on plasma HIV RNA concentration as discussed by Volberding and Deeks [26]. Although CD4 count has also been used to monitor ART, recent studies suggest that it may not detect early treatment failure adequately as discussed by Moore et al. [27]. POC test for CD4 count could help clinicians in resource limited settings to decide when to start antiretroviral treatment, and a POC test for viral load would be of great value in identifying treatment failure and the need for second-line treatment. POC devices for CD4 immunologic monitoring and viral load assay are currently being evaluated as discussed elsewhere [28–30]. If validated, these devices could rapidly and accurately identify CD4 counts with minimal operator training, infrastructural setup, and with less cost than standard laboratory-based equipment such as flow cytometers for CD4 count or RT-PCR for viral load assay. PIMA analyzer (Alere, Inc., Waltham, MA, USA) is a WHO prequalified simple, effective point-of-care CD4 count test. It gives a CD4 count in 20 minutes from a finger stick or venous sample. Recent evaluations in Zimbabwe and Mozambique have shown good performance in comparison to flow cytometry as discussed elsewhere [27, 28]. VISITECT CD4 is a disposable, semiquantitative point-of-care rapid test for the determination of CD4 counts in whole blood. VISITECT CD4 can guide treatment decisions at the point-of-care, without the need for extensive training or sophisticated equipment. The test is a convenient solution for use in laboratories and remote clinics worldwide and provides a visual “TREAT” or “NO TREAT” result within 40 minutes [31].

Antiretroviral drugs, especially stavudine (nucleoside reverse transcriptase inhibitors), are associated with severe side effects such as lactic acidosis, pancreatitis, and hepatitis as discussed by Arora et al. [32]. POC tests for toxicity monitoring (e.g., lactate, renal function tests, and liver function tests) are also being evaluated as discussed elsewhere [33, 34] and will help in monitoring drug toxicity in patients. Tuberculosis is one of the most common opportunistic infection in developing countries as discussed by Arora et al. [35]. Therefore, there will also be a need for rapid POC detection of opportunistic infections. 

## 7. Conclusion

Decentralization of laboratory services is required to detect the maximum possible number of HIV-positive patients and to put them on antiretroviral therapy. Accurate HIV rapid test devices are available but are not yet widely used. They may improve patient care especially in outreach settings for hard to reach groups and in obstetric management. The particular public health interest in the use of point-of-care testing for HIV is its ability to contribute to health goals which include preventing new HIV infections, reducing the number of HIV individuals who are unaware of their status, and promoting linkage of HIV-positive individuals to care. The ethical framework surrounding informed consent for a rapid test is the same as for a standard blood test, but the dissemination of testing and potential lack of experience of staff administering tests and handling the results requires careful consideration.
